# Serous labyrinthitis as a manifestation of cat scratch disease: a case report

**DOI:** 10.4076/1752-1947-3-7405

**Published:** 2009-09-15

**Authors:** Ilias Kantas, Michael Katotomichelakis, Marinos Vafiadis, Zografia V Kaloutsa, Chariton E Papadakis

**Affiliations:** 1 Department of Otolaryngology, 'G. Genimmatas' General Hospital, Karditsa, Thessaloniki, Greece; 2 Department of Otolaryngology, 'Agia Olga' General Hospital, Athens, Greece; 3 Department of Otolaryngology, Chania General Hospital, Chania, Crete, Greece

## Abstract

**Introduction:**

Cat scratch disease is an infectious disease transmitted by young cats, in which the principal causative factor is *Bartonella henselae*. The typical course of cat scratch disease is usually benign and self-limited and requires only supportive therapy. However, cases lasting up to 2 years have been reported, and more serious complications may occur. Many manifestations of the disease have been reported by different medical disciplines.

**Case presentation:**

A case of cat scratch disease in a 71-year-old Greek woman with an unusual clinical course is presented here. Serous otitis media was combined with rotational vertigo due to labyrinthitis. The invaded ear was ipsilateral to the inoculation site.

**Conclusion:**

Cervicofacial lymphadenopathy has been demonstrated as the most common otolaryngologic manifestation of cat scratch disease. Manifestation in the middle and inner ear has, to the best of our knowledge, not been reported before. Our report presents a patient with cat scratch disease with clinical signs and symptoms in the middle and inner ear.

## Introduction

Selby and Walker advocated an avasculitic origin of cat scratch disease (CSD) after demonstrating cerebral arteritis in a 7-year-old girl suffering from CSD [1]. In 1992, Regnery *et al.* reported *Bartonella henselae* to be the major causative agent of CSD [2]. Therefore, CSD is considered as a zoonosis with many clinical manifestations as well as a cosmopolitan emerging human pathogen [3]. Typical CSD can occur in all ages. Usually, 3 to 10 days after contact with an infected cat, most often a newly acquired kitten, a non-tender 1 mm to 10 mm red to brown papule develops, followed by prolonged regional lymphadenopathy for another 14 days.

Most authors have described typical CSD as a self-limited disease with a subsidence of symptoms within 2 to 4 months. However, cases lasting up to 2 years have been reported, and more serious complications may occur. Different organs can be affected, including the eyes, liver, spleen, central nervous system, skin and bones, among others [3,4]. Many otolaryngologic manifestations of the disease have been observed. However, manifestations in the middle or inner ear have, to the best of our knowledge, not been reported so far. Our report presents a patient with CSD with clinical signs and symptoms in the middle and inner ear.

## Case presentation

A 71-year-old Greek woman complaining of rotational vertigo was admitted to the ENT (ear, nose and throat) department at the "G. Genimmatas" General Hospital of Thessaloniki, for investigation and treatment. The patient had an unexplained fever, with an axillary body temperature of over 39°C, taken in several measurements during the 15 days before admission.

During ENT clinical examination and Romberg's test, the patient tended to fall towards the right. Observation using Frenzel glasses showed spontaneous nystagmus with the fast component toward the left.

**Figure 1:**
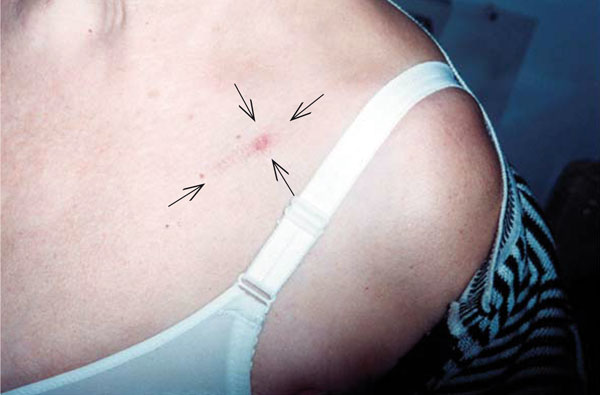
**A skin lesion resembling a round, red to brown, non-tender papule on the left subclavicular area**.

Otomicroscopic examination revealed serous otitis media on the left ear. ENT endoscopic examination for rhinopharyngeal, oropharyngeal or hypopharyngeal lesions was negative. Neurologic examination was normal. A block of the axillary lymph nodes, not fluctuant or accompanied by overlying erythema, was presented on the left side. A skin lesion resembling a round, red to brown, non-tender papule was observed on the left subclavicular area during clinical examination (Figure 1). The patient did not report any previous history of influenza or virus infection. However, she mentioned having daily contact with two kittens. This history, along with the skin lesion on the left subclavicular area, raised the suspicion for CSD, although the patient did not remember any cat scratching or biting.

Rinne's test was negative on the left and positive on the right ear, respectively. The patient localized the tone on the left side during Weber's test. Tympanometry appeared as a flat curve on the left side (a type B tympanogram) due to serous exudate in the middle ear. The right-ear tympanogram showed a normal type A curve. A pure-tone audiogram showed a conductive hearing loss on the left side with a gap of 10 dB to 15 dB. On electronystagmography testing, the main abnormal finding was the presence of an intense spontaneous nystagmus of a peripheral type, both with and without an optic fixation. The direction of the fast phase of the nystagmus toward the site of the lesion along with the presence of a directional preponderance (83%) towards the side of the lesion showed an impaired horizontal semicircular canal of the left labyrinth.

Differential diagnosis for specific lymphadenopathy includes tuberculosis, sarcoidosis, syphilis, diphtheria, tularemia, toxoplasmosis and CSD. The specific history and the skin lesion pointed towards CSD. The chest radiogram was normal. Erythrocyte sedimentation rate was 78 mm per hour, and C-reactive protein was 56 mg/dL. Immunoglobin (Ig) M and IgG serologic control was negative for toxoplasmosis, cytomegalovirus, Epstein-Barr virus and HIV.

Serologic control for *Bartonella henselae* was sent to National Institute of Pasteur in Athens. Azithromycin was administered for 6 days in combination with methylprednisolone and dimenhydrinate during the wait for the test results, which confirmed the diagnosis of CSD. Paracentesis of the tympanic membrane was performed, and 2 cc of fluid materials from the mesotympanum were taken and sent for culture tests. After wide tympanostomy, mucosal pieces were taken from the promontory area using micro ear forceps with oval cupped jaws and sent for polymerase chain reaction (PCR) control. Culture results were negative for *Bartonella henselae*. Total DNA was isolated from promontory mucosal cells, according to the standard procedures (QIAGEN-Tissue DNA isolation kit, QIAGEN GmbH, Germany) and was used as a template in a real-time PCR (ROCHE LightCycler 1.1), with primers and probes (FRET technology) specially designed to multiply and detect the *OspA* region of *Bartonella* species (*B. bacilliformis, B. elizabethae, B. vinsonii, B. quintana* and* B. henselae*). The results obtained by the PCR analysis revealed the presence of *Bartonella henselae* DNA in the clinical specimen. The first serologic results for *Bartonella henselae* strengthened our suspicion for CSD (IgM: 20, IgG: 270). IgG blood antibodies increased impressively 3 weeks later (IgG: 1100). This four-fold rise of IgG blood antibodies finally confirmed the diagnosis of CSD. The patient was discharged from the hospital symptom-free one week after admission.

## Discussion

Epidemiologic studies revealed that owners of kittens younger than 12 months were 15 times more likely to develop CSD than owners of adult cats [5]. Typical CSD can occur in immunocompromised or immunocompetent patients of all ages alike. The history is always associated with cat scratches, bites or exposure to cat saliva. The disease initially develops as a small skin lesion at the site of the scratch or bite, followed by a round red to brown, non-tender papule after 3 to 10 days [6].

*Bartonella henselae* is a proteobacteria that can cause bacteremia, endocarditis, bacillary angiomatosis, peliosis hepatis and CSD. The diagnosis of CSD is usually confirmed by the presence of IgM antibodies against *B.henselae* after serologic control and in combination with IgG antibodies at the first examination, which then shows a rising titer in a second serologic follow-up after 10 to 14 days [7]. When it is not possible to isolate the causative agent within the labyrinthine fluids, identification of the agent within the tissue of the middle ear is almost as reliable [8]. The histologic findings of invaded lymph nodes are inflammatory granulomas with micro-abscesses surrounded by histocytes, lymphocytes, occasional giant cells and fibrosis. In other words, they are very similar to the findings in other granulomatous diseases such as tularemia, lymphogranuloma venereum and brucellosis and have to be distinguished from these by molecular methods [7].

CSD may present in a typical or atypical fashion in both immunocompetent and immunocompromised patients. The spectrum of CSD includes lymphadenopathy, usually regional and unilateral, which occurs 1-3 weeks after the infecting cat scratch, with gradual nodal enlargement. Sometimes, patients appear to be quite well generally, with only mild, non-specific symptoms such as headache, anorexia, myalgia or abdominal pain [9].

The most typical manifestation of CSD is Parinaud's oculoglandular syndrome, consisting of unilateral preauricular lymphadenopathy and conjunctivitis [9]. Unexplained fever, Keber's stellate neuroretinitis [9], endocarditis [10], encephalopathy [11], acute hemiplegia [6], facial palsy and partial ptosis [12] have all been reported as manifestations of CSD. Ridder *et al*. [7] described the most usual ENT manifestations of CSD in a number of the 99 patients they examined, after serologic confirmation in a retrospective analysis. These authors reported lymphadenopathy in 100% of cases as the first manifestation (acute, abscessed and chronic), followed by a swelling of the parotid lymph nodes (34.3%), pharyngitis (15.2%), fever (9.1%), Parinaud's syndrome (8.1%) and swelling of parotid gland (6.1%). As rare manifestations, they reported tonsillitis, laryngitis, and perichondritis of the auricle [7]. The same first author reported a case of CSD mimicking a hypopharyngeal tumor [13].

Azithromycin has been advocated as modestly hastening the resolution of lymphadenopathy [14]. Macrolides, rifampin, doxycycline, gentamicin, trimethoprim-sulfamethoxazole and ciproxin alone or in combination have also been suggested as agents for treatment.

The pathogenesis of CSD complications in the central nervous system still remains obscure. To the best of our knowledge, manifestation in the inner and middle ear have not been reported so far. The pathogenesis of serous labyrinthitis in our patient may be due to toxins present in the labyrinth, rather than due to the presence of bacteria in the inner ear.

## Conclusion

The manifestation of CSD in the middle and inner ear is rare.

## Consent

Written informed consent was obtained from the patient of this case report for publication and any accompanying images. A copy of the written consent is available for review by the Editor-in-Chief of this journal.

## Competing interests

The authors declare that they have no competing interests.

## Authors' contributions

IK conceived the study, and was involved in drafting the manuscript. MK participated in the study design, and was involved in drafting the manuscript. MV and ZVK collected the data, formatted the images, participated in the study design and were involved in drafting the manuscript. CEP participated in the study design and coordination, performed language corrections and critically evaluated the article. All authors read and approved the final manuscript.

## References

[B1] Selby G Walker GL Cerebral arteritis in cat-scratch disease Neurology 1979 29 1413 1418 573385 10.1212/wnl.29.10.1413

[B2] Regnery RL Olson JG Perkins BA Bibb W Serological response to "Rochalimaea henselae" antigen in suspected cat-scratch disease Lancet 1992 339 1443 1445 10.1016/0140-6736(92)92032-B 1351130

[B3] Anderson BE Neuman MA Bartonella spp as emerging human pathogens Clin Microbiol Rev 1997 10 203 219 9105751 10.1128/cmr.10.2.203 PMC172916

[B4] Bass JW Vincet JM Person DA The expanding spectrum of Bartonella infections: II Pediatr Infect Dis J 1997 16 163 179 10.1097/00006454-199702000-00002 9041596

[B5] Zangwill KM Hamilton DH Perkins BA Regnery RL Plikaytis BD Hadler JL Cartter ML Wenger JD Cat scratch disease in Connecticut. Epidemiology risk factors and evaluation of a new diagnostic test N Engl J Med 1993 329 8 13 10.1056/NEJM199307013290102 8505963

[B6] Rocha JL Pellegrino LN Riella LV Martins LT Acute hemiplegia associated with cat-scratch disease Brazil J Infect Dis 2004 8 263 266 10.1590/S1413-86702004000300012 15476060

[B7] Ridder GJ Boedeker CC Technau-Ihling K Sander A Cat-scratch disease: otolaryngologic manifestations and management Otolaryngol Head Neck Surg 2005 132 353 358 10.1016/j.otohns.2004.09.019 15746844

[B8] Davis LE Johnsson LG Viral infections of the inner ear: clinical, virologic, and pathologic studies in humans and animals Am J Otolaryngol 1983 4 347 362 10.1016/S0196-0709(83)80022-2 6314834

[B9] Batts S Demers DM Spectrum and treatment of cat scratch disease Pediatr Infect Dis J 2004 23 1161 1162 15626957

[B10] Baorto E Payne RM Slater LN Lopez F Relman DA Min KW St Geme 3rd Culture-negative endocarditis caused by Bartonella Henselae J Pediatr 1998 132 1051 1054 10.1016/S0022-3476(98)70410-X 9627605

[B11] Noah DL Bresee JS Gorensek MJ Rooney JA Cresanta JL Regnery RL Wong J del Toro J Olson JG Childs JE Cluster of five children with acute encephalopathy associated with cat-scratch disease in south Florida Pediatr Infect Dis J 1995 14 866 869 10.1097/00006454-199510000-00009 8584313

[B12] Ganesan K Mizen K Cat-scratch disease an unusual cause of facial palsy and partial ptosis: case report J Oral Maxillofac Surg 2005 63 869 872 10.1016/j.joms.2004.05.222 15944991

[B13] Ridder GJ Richter B Laszing R A farmer with a lump in his throat Lancet 1998 351 954 9734943 10.1016/S0140-6736(05)60607-1

[B14] Bass JW Freitas BC Freitas AD Sisler CL Chan DS Vincent JM Person DA Claybaugh JR Wittler RR Weisse ME Regnery RL Slater LN Prospective randomized prospective randomized double blind placebo-controlled evaluation of azithromycin for treatment of cat-scratch disease Pediatr Infect Dis J 1998 17 447 452 10.1097/00006454-199806000-00002 9655532

